# Ghostwriters in the scientific world

**DOI:** 10.11604/pamj.2018.30.217.16312

**Published:** 2018-07-18

**Authors:** Sankalp Yadav, Gautam Rawal

**Affiliations:** 1 General Duty Medical Officer-II, Department of Medicine & TB, Chest Clinic Moti Nagar, New Delhi, India; 2 Associate Consultant, Department, of Respiratory Intensive Care, Max Super Specialty Hospital, New Delhi, India

**Keywords:** Drug, ghostwriters, scientific publishing

## Abstract

The scientific world is facing a constant problem of ghostwriters. These ghostwriters are often attached to the medical publishing houses and are involved in writing an article for a pharmaceutical company which may, in turn, use the name of an established or a famous scientist as an author to the article. Often, such articles are published in well-known journals and are circulating widely. Many a time the adverse effects are overlooked in such papers. This will result in a corrupt practice of prescribing a drug which is not scientifically useful or may have life-threatening side effects. In this present article, the authors discuss this evil practice of ghostwriting in the context of the present day scientific publishing.

## Introduction

The scientific writing is an important part of the skill and career development [1]. The scientific world is full of medical journal publishing articles that lay the foundation of the future practice of medicine. However, in the context of this, there is a continuing problem of ghostwriting [2]. The term ghost authorship means that an individual who contributed substantially to a manuscript is not named in the byline or acknowledgments [2,3]. The ghostwriters came after the development of professional medical writers in the late 20^th^ century [4]. There are thousands of such medical/ghost writers around the globe [4]. The international organizations like the International Committee for Medical Journal Editors (ICMJE) do not even acknowledge their existence [4]. As per the European Medical Writers Association (EMWA), the ghostwriter is the one who prepares a manuscript for an author, but the writer is not given an authorship [5]. The whole concept of ghostwriting could have been based on the need for the conversion of the raw data to an intelligible document [4]. However, it has been abused to a greater extent and even established biomedical journals are not spared [4,6]. A study reported that even the Cochrane Reviews has 39% of authors who do not qualify to be the real authors and do not match the authorship guidelines set by the ICJME and of this 11% were ghost authors [7,8]. In this paper the authors, highlight the issues associated with ghostwriting.

## Methods

The authors searched the term “ghost author”, “ghostwriter” and “ghost authorship” on Google and read the scholarly articles. Besides, a similar search was made on the PubMed to study the gravity of this problem looming large in the scientific world. A detailed overview of this malpractice and possible solutions are suggested in this paper.

## Results

The extent of ghostwriting is very wide. The ghostwritten industry-sponsored articles are a common unethical practice and continue to plague biomedical journals which publish industry-sponsored research [9-12]. Healy reported that the major amount of pharmaceutical articles was ghostwritten [13]. Flanagin et al. 1998, reported that a substantial proportion of articles (up to 11%) in six peer-reviewed medical journals demonstrate evidence of ghost authors [14]. Stretton 2014, in her paper, reported the details of six cross-sectional surveys in which the prevalence of possible ghostwriting varied from 0.9% to 24.1% [12,14-18]. Also, four cross-sectional survey publications reported the prevalence of ghostwriting/author varied from 0.7% to 70% of publications or authors [12]. As per the recent studies in the British Journal of Psychiatry and the JAMA, around 11% to 50% of the articles on pharmaceuticals that appear in the major biomedical journals may actually be ghostwritten [19,20]. In a detailed study Gøtzsche et al 2007, reported the extent to be around 75% [21].

## Discussion

**Why ghostwriting**? The extent of ghostwriting is very large [22]. The majority of the scientific data that becomes available due to a large number of researches going in the various fields needs to be converted into a presentable scientific document [23]. One way to do so is by the use of professional medical writers who ethically help the researchers to bring the raw data into an intelligible document [23]. The other is by Medical Education and Communications Company or MECC, that includes a number of professional experts in the field of scientific writing and editing [23,24]. These MECC is industry funded and is paid to write articles in favor of the products of the company [24]. However, if the legitimate services of medical writing agencies or independent writers are used for the vested interest in order to write something for a product or drug overlooking the adverse effects then this constitutes the ghostwriting [23]. Many times the ghostwriters are funded by the big pharmaceutical companies [24]. The main principle for this practice is that the pharmaceutical companies fund the ghostwriters to prepare the manuscript which is forwarded to an established scientist in the field who may or may not be allowed to make changes and in turn, is sent to a reputed journal for publication [24]. The expert scientist is sometimes not even aware of the funding for this malpractice [22]. These industry-funded ghostwritten articles are aimed to bring the positive side of the drug and many a time overlooking the adverse effects of the drug [24]. The reputed journals are often chosen, as this brings credibility to the scientific data in the paper [22]. The higher the repute of the journal bigger will be its audience and higher will be the number of citations, thus adding weight to the actual paper [22]. The manuscript published is circulated all over the world and in this way the pharmaceutical companies make huge profits from the sales of the drug recommended by an expert in a reputed journal [24]. It has been well reported in the medical literature that the ghostwriters have been paid huge funds [22,25].

**Some famous examples**: The grave problem of ghostwriting is evident by really famous examples like the marketing of drugs Fen-Phen, SSRIs, Neurontin, Zoloft, Redux, Vioxx, etc [22,25,26]. The manuscripts which were published on these drugs were ghostwritten and the side-effects were totally ignored [22]. The ghostwritten papers were in large quantities, thus ensuring a favorable outcome in support of the investigational drug even if the efficacy of the drug is reviewed by a meta-analysis [22]. The actual problem with the whole process of ghostwriting is grave because of non-transparency in the data presented [13]. Resulting in serious ramifications on the public health [24]. The ghostwriting has been also considered as an institutionalized plagiarism [22]. However, there are guidelines which recommend that anybody involved in the plagiarism should be subjected to strict disciplinary action [22]. These punishments could even involve the retraction of the paper [1]. But only a small number of all the retracted articles are ever annulled due to plagiarism [1]. The EMWA recommends that the authors of the biomedical papers should be in full control of the papers even if the same is written by an expert medical writer [5]. The role of medical writers should be clearly defined and mentioned either as an author in a review paper (if a substantial contribution is there and willingness to share responsibility is present) or in the acknowledgements or the contributors' section [5,27-30]. The interaction between the professional writers and the real authors of a manuscript should be clearly defined using the GATE principles based on the Guarantee, Advice, Transparency, and Expertise as defined elsewhere [23,28]. But the huge amount of funds involved in the ghostwriting and the same provided to the experts just for the sake of taking their name for a biased research, usually funded by a big industry is resulting in dubious data being published in reputed journals thereby putting the public health in jeopardy [24]. The importance of ethical authorship and good publication practices is extremely important [31]. The malpractice of ghostwriting is not only present in the pharmaceutical articles, but the same is present in academia as well [31]. The journals and the medical publishing houses have to take radical steps to curb this problem of ghostwriting [23]. The industries that fund these will never control it [22]. The peer-reviewed data are an important part of making health care paradigms and thus the false information will lead to serious consequences on the public health [2]. The impact could be devastating, especially in the developing countries with low expenditure on health and where the per capita income is low and where the problem of corruption is very big [32,33].

**The good aspects of professional medical writing**: There is a small but clear line of demarcation between a professional medical writer and a ghostwriter [23]. Although the ghostwriting is having serious impacts on the science, there are some good points in favor of an ethical medical writer, if appropriately acknowledged [19]. First, an assisted scientific writing brings the results of the research to the public which, if left to senior clinicians would never be reported due to their busy schedule [23,19]. Also, it has been observed that the senior clinicians may not be familiar with the details of the literature to produce a timely and comprehensive text [23,28]. Secondly, the overall paper quality will be superior due to professional writing as the medical writers are the specialist in writing a manuscript [23,19]. Third, based on the scientific data the adverse drug reactions could well be reported comprehensively in an industry-sponsored research [19,34]. Fourth, that some professional writing agencies may disclose the conflicts of interest more clearly [19]. Fifth, the clinicians, especially from developing countries have a huge burden of clinical works in addition to the research work and thus the services of professional medical writers will help with the publication of their work and thus will be made available to the general public [23]. This is also supported by the fact that every year the reputed journals return several important manuscripts for revision or even reject them due to poor writing styles, poor organization of the content and even poor English and grammar [23,35,36]. Sixth, the professional writers bring clarity to the articles as if the same may be lacking, then the actual idea that the authors want to communicate may be lost [23, 37,38]. The professional medical writers are legitimate contributors to the article [39,40]. They are a blessing while ghostwriters are intellectually dishonest, unethical and a curse [41].

## Conclusion

To control this problem, a group of medical writers developed the EMWA [42]. The purpose of EMWA is to identify the issues with the malpractice of ghostwriting in scientific literature and also to suggest the ways to curb it [42]. The ghostwriting can be controlled if the stakeholders like journals, authors, medical writing agencies and the agencies involved in supervising the publications work in unison [23,43]. At any stage of the peer review if any, involvement of ghostwriter is suspected by the reviewers then the same should be reported to the editors [23]. The editors must ensure that a contributorship statement is submitted by the authors [23]. The section for instruction to authors should include guidance for authorship and acknowledgement [23]. The ICJME has published important criteria for authorship and also the World Association of Medical Editors (WAME) developed a specific policy on ghostwriting [41,44]. Besides, the function of the medical writer in preparing biomedical journal manuscripts is of interest to task forces convened by American Medical Writers Association (AMWA) and EMWA and has resulted in articles of guidelines for medical writers in preparing the scientific documents [4]. Even after the presence of guidelines, it is very difficult to identify any involvement of an industry in the preparation of any manuscript. Also, if the industry has funded the research and has hired the writing agency then the published paper may become more authentic if the raw data on which the paper is based is also made available to the scientific community for an unbiased analysis and interpretation [45]. The ghostwriting has really gone to the roots of scientific research and efforts to control it are imperative.

### What is known about this topic

- Ghostwriting is prevalent in scientific literature and is underreported;
- Large publication houses are also affected;
- Reputed researchers are the target.

### What this study adds

- The extent of ghostwriting is between 0.9%-75%;
- A substantial amount of scientific papers are ghostwritten, policy changes need to include strategies to reduce ghost authorship, in order to curb the overall extent of ghostwriting;
- Emphasis on the development of guidelines similar to those developed by European Medical Writers Association (EMWA), throughout the world to control the malpractice of ghostwriting in scientific literature.

## Competing interests

The authors declare no competing interests.
